# Supplementary material for: FCRLs and atypical transcriptional pattern in tumor infiltrating B cells from lung and renal cancer
Source: Front Immunol. 2025 Sep 8;16:1587088. doi: 10.3389/fimmu.2025.1587088 (PMC12450953; doi:10.3389/fimmu.2025.1587088)
Supplement: Supplementary Table 1 — Patient characteristics - lung cancer. [file Table1.pdf]

Supplementary table 1. Patient characteristics - lung cancer

№	patient ID	histology	stage
1	lc-p7	adenocarcinoma	T3N0M0
2	lc-p8	squamous cell carcinoma	T3N0M0
3	lc-p9	adenocarcinoma	pT2N3M0
4	lc-p12	adenocarcinoma	T2aN0M0
5	lc-p15	adenocarcinoma	T2aN0M0
6	lc-p16	adenocarcinoma	T2aN0M0
7	lc-p17	adenocarcinoma	T1N1M0
8	lc-p20	adenocarcinoma	T1BN0M0

Supplementary table 2. Patient characteristics - renal cancer

**№**	**patient ID**	**histology**	**stage**
1	rc-p12	renal cell clear cell carcinoma	cT3cNxM1
2	rc-p16	renal cell clear cell carcinoma	cT3aNxM0
3	rc-p17	renal cell clear cell carcinoma	no information
4	rc-p18	renal cell clear cell carcinoma	cT3bN0M0
5	rc-p19	renal cell clear cell carcinoma	cT3cNxMx
6	rc-p20	renal cell clear cell carcinoma	cT2N1Mo
7	rc-p21	renal cell clear cell carcinoma	no information
8	rc-p22	renal cell clear cell carcinoma	pT3aN0M0
9	rc-p23	renal cell clear cell carcinoma	T3cNxM0
10	rc-p25	renal cell clear cell carcinoma	cT2N0M1
11	rc-p26	renal cell clear cell carcinoma	cT3N0M0
