# Supplementary material for: FCRLs and atypical transcriptional pattern in tumor infiltrating B cells from lung and renal cancer
Source: Front Immunol. 2025 Sep 8;16:1587088. doi: 10.3389/fimmu.2025.1587088 (PMC12450953; doi:10.3389/fimmu.2025.1587088)
Supplement: Supplementary Figure 1 — (A) Diagram representing the scRNAseq data analysis steps. (B) Histogram of clusters split by sample of origin to assess the efficiency of batch effect removal. (C) Dotplot of B-cell subpopulation marker gene expression across 13 Bmem clusters. (D) Histogram of the IGHA1/IGHG1 isotype expression across the clusters. The cells with the zero expression of one of IGHA1/IGHG1 were considered single-positive. (E) B cell subpopulation marker gene expression mapped to UMAP representation of Bmem from LUAD tumors.. [file Image1.pdf]

Supplementary Figure 1.

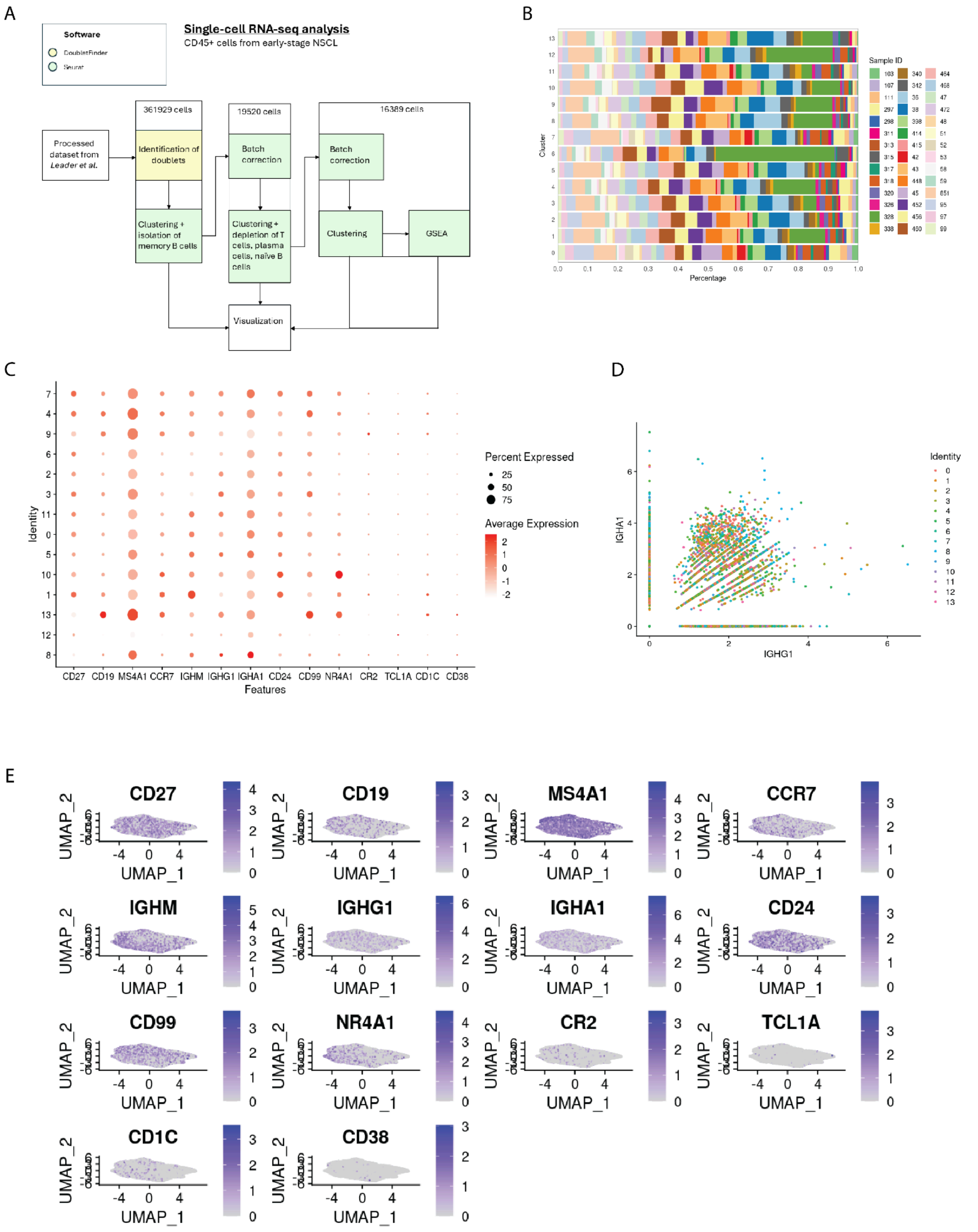

Supplementary Figure 1. A - Diagram representing the scRNAseq data analysis steps. B - Histogram of clusters split by sample of origin to assess the efficiency of batch effect removal. C - Dotplot of B-cell subpopulation marker gene expression across 13 Bmem clusters. D - Histogram of the IGHA1/IGHG1 isotype expression across the clusters. The cells with the zero expression of one of IGHA1/IGHG1 were considered single-positive. E - B cell subpopulation marker gene expression mapped to UMAP representation of Bmem from LUAD tumors.

Supplementary Fig. 2

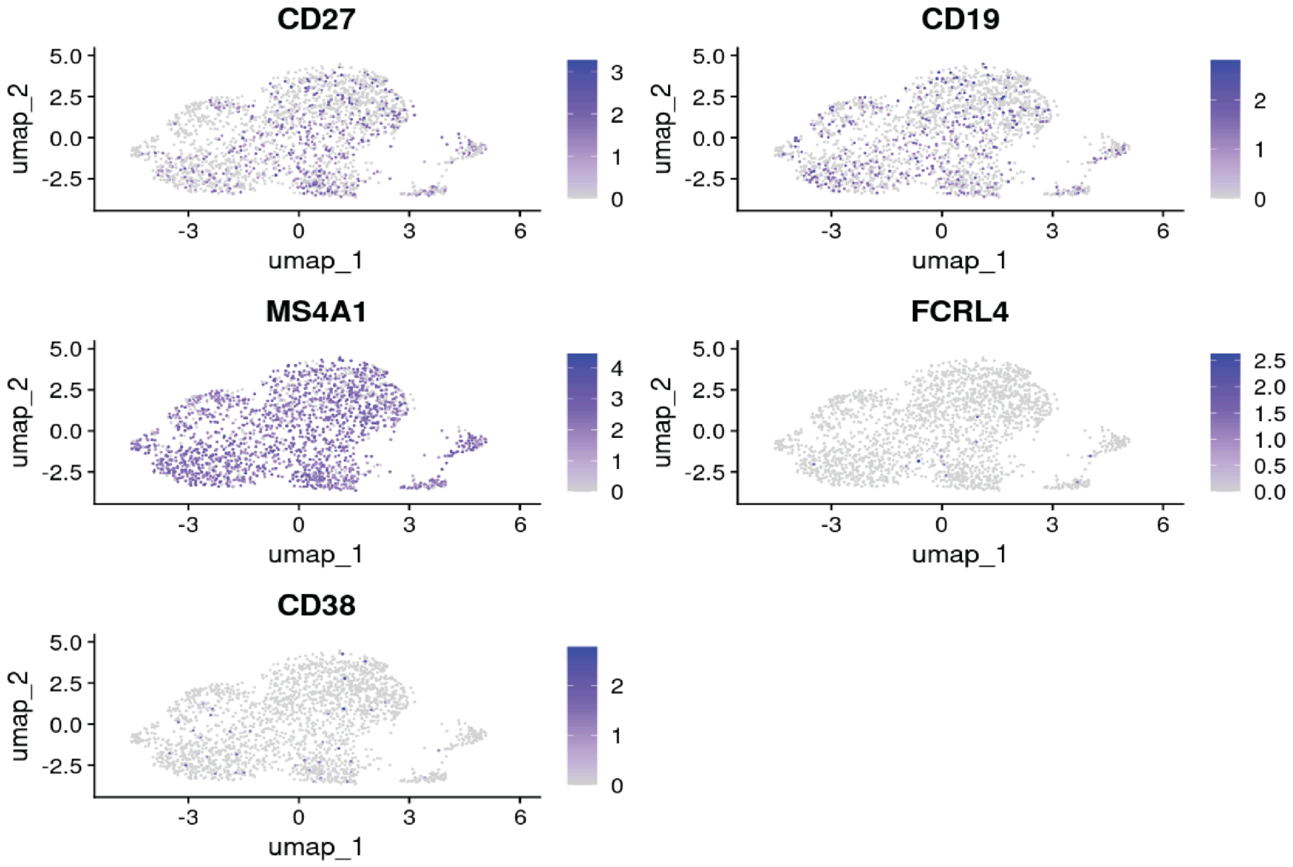

Supplementary Figure 2. Expression of major genes of interest mapped to the UMAP representation of memory B cells from the normal lung tissue.
